# First detection and phylogenetic analysis of porcine circovirus 3 in female donkeys with reproductive disorders

**DOI:** 10.1186/s12917-021-03013-6

**Published:** 2021-09-18

**Authors:** Tongtong Wang, Wenqiong Chai, Yonghui Wang, Wenqiang Liu, Zetong Huang, Li Chen, Ruihong Guo, Yuting Dong, Mengyuan Liu, Qiankun Zheng, Guiqin Liu, Changfa Wang, Wen-Ping Guo, Sidang Liu, Liangliang Li

**Affiliations:** 1 Research Institute of Donkey High-efficiency Breeding and Ecological Feeding, College of Agronomy, Liaocheng University, Liaocheng, China; 2 Delisi Group Co., LTD, Zhucheng, China; 3 Department of Pathogenic Biology, College of Basic Medicine, Chengde Medical University, Chengde, China; 4 College of Animal Science and Technology, Shandong Agricultural University, Tai’an, China

**Keywords:** Donkeys, Porcine circovirus 3, Prevalence, Phylogenetic analysis

## Abstract

**Background:**

PCV3 is a pathogen associated with porcine dermatitis and nephropathy syndrome (PDNS)-like clinical signs, reproductive failure, and cardiac and multiorgan inflammation, which was newly identified in 2016 in sows in USA. Recently, PCV3 has also been identified from several non-porcine species like (cattle, dog, wild boar, deer, mice and ticks). However, PCV3 infection in donkey is not well established. Since 2019, 300 blood samples were collected from female donkey, which was characterized by abortion and sterility, in Liaocheng city of China.

**Results:**

In the present study, an investigation of PCV3 in donkey blood samples was undertaken employing by real time PCR. Positive rates of PCV3 in donkeys reach to 21.0 %. In addition, one full-length PCV3 genome sequence was obtained, and it had a highest identity with porcine circovirus 3 PCV3/CN/Nanjing2017 strain and is clustered to PCV3a genotype based on ORF2 sequences.

**Conclusions:**

This is the first report of detection of PCV3 from female donkeys presenting reproductive failure in large-scale donkey farms, China. In addition, the PCV3 strain identified in this study shared the closest relationship with those from porcine, suggesting that PCV3 may be transmitted from pigs to donkeys. Totally, PCV3 infection in donkey should be concerned although the association between it and reproductive failure are not better understood.

**Supplementary Information:**

The online version contains supplementary material available at 10.1186/s12917-021-03013-6.

## Background

Porcine circovirus 3 (PCV3), a new member of the genus *Circovirus* in the family *Circoviridae*, is characterized by a circular, single-stranded DNA genome [1, 2]. To date, PCV3 has been found in several species, such as dogs, cattle, mice, ticks, wild boar, deer, even baboons infected by trans-species transmission by transplantation of a heart from a PCV3-positive donor pig [3–6]. In swine, PCV3 cases have been associated with porcine dermatitis and nephropathy syndrome (PDNS), multi systemic inflammation, and reproductive failure [7, 8]. Moreover, the high prevalence of PCV3 in pigs and in wild boars without specific clinical signs has been also reported [9–12]. However, the knowledge about the infection of PCV3 in donkeys is not well understood.

In the present study, 300 blood samples from female donkeys with reproductive failure from Liaocheng city, China, were tested by real time PCR for PCV3 as described previously [13]. The percentage of PCV3 infection in donkeys was 21.0 %. The genetic analysis of donkey-origin PCV3 shared the closest relationship with PCV3/CN/Nanjing2017strain (MK580468.1). Therefore, further research is warranted to investigate the potential of PCV3 to cause clinical disease in mares. Additionally, donkeys may serve as reservoirs for the virus, adding to the complex and poorly understood infection dynamics between pigs and other species.

## Results

### PCV3 detection

A total of 300 blood samples from 5 different donkey farms were tested for PCV3 using real time PCR. These results demonstrated that 21 % (63/300) of the blood samples were PCV3 positive. Specifically, 24.7 % (21/85) in Chiping county, 7.5 % (4/53) in Dong’e county, 17.0 % (8/47) in Gaotang county, 31.7 % (19/60) in development zone, 20.0 % (11/55) in high-tech zone. The copies number of PCV3 in blood was calculated in Table S1. These results suggested the prevalence of PCV3 in donkey farms in Liaocheng city, China.

### Genome sequencing of donkey-origin PCV3 strain

The fragments of PCV3 complete genome sequences were amplified as described previously [14]. After sequence assembling, the complete genome of the PCV3 from donkey named as PCV3/CN/SD-DK was 2000 nucleotides in length similar to other PCV3 strains. In detail, two ORFs, ORF1 (891 nucleotides) and ORF2 (645 nucleotides), were found, and they encoded the deduced Rep protein and capsid protein, respectively. The full-length PCV3/CN/SD-DK sequence was deposited into the GenBank database under accession numbers MW715784.

### Multiple sequences comparison and phylogenetic analyses

**Fig. 1:**
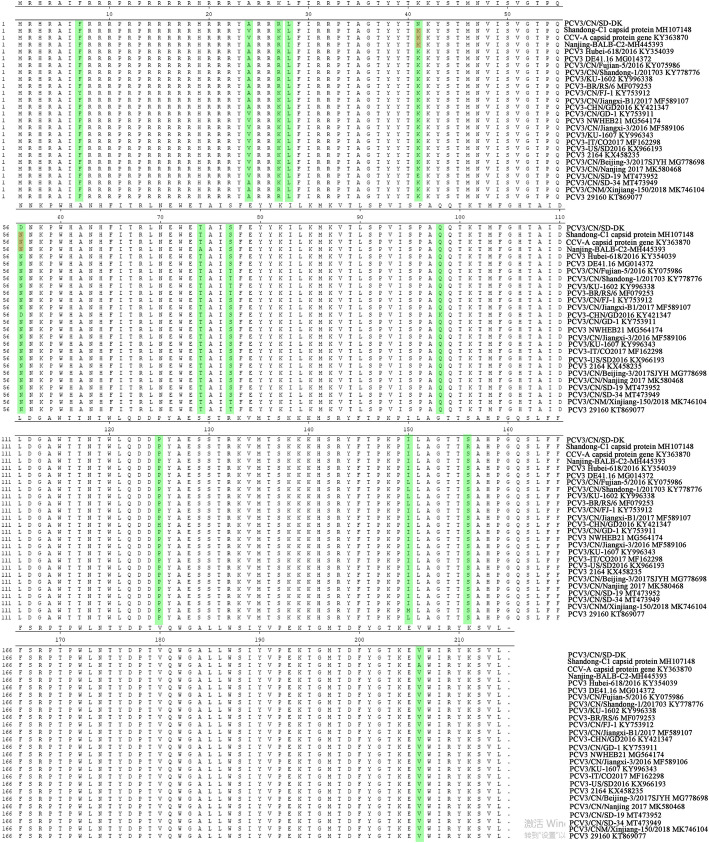
Alignment of amino acid sequences of Cap between donkey with that of other species PCV3 (dog, cattle, mice and pig)

In the current study, the complete genome sequence of PCV3/CN/SD-DK strain (indicated in additional file 1) showed 99.3 % identity to PCV3/CN/Nanjing2017, and presented 97.9-99.2 % identity with other PCV3 sequences. In the case of PCV3/CN/SD-DK, ORF1 shared 98.8-99.6 % nucleotide similarity with other PCV3 ORF1, and 97.0-99.0 % based on the deduced amino acid sequences. However, its ORF2 gene is highly variable, shared 98.3-98.9 % nucleotide homology with other PCV3 ORF2, and 96.7–98.6 % at amino acid level. PCV3 Cap proteins from dog (CCV-A capsid protein gene KY363870), cattle (Shandong-C1 capsid protein gene MH107148) and mice (Nanjing-BALB-C2-MH445393) have the same discrepancy at aa residues 41 and 56 position with that of PCV3/CN/SD-DK (Fig. 1, marked in red). In addition, there are 13 positions difference between PCV3/CN/SD-DK and other variants from other species rather than donkey in Cap protein (Fig. 1, marked in green).

**Fig. 2:**
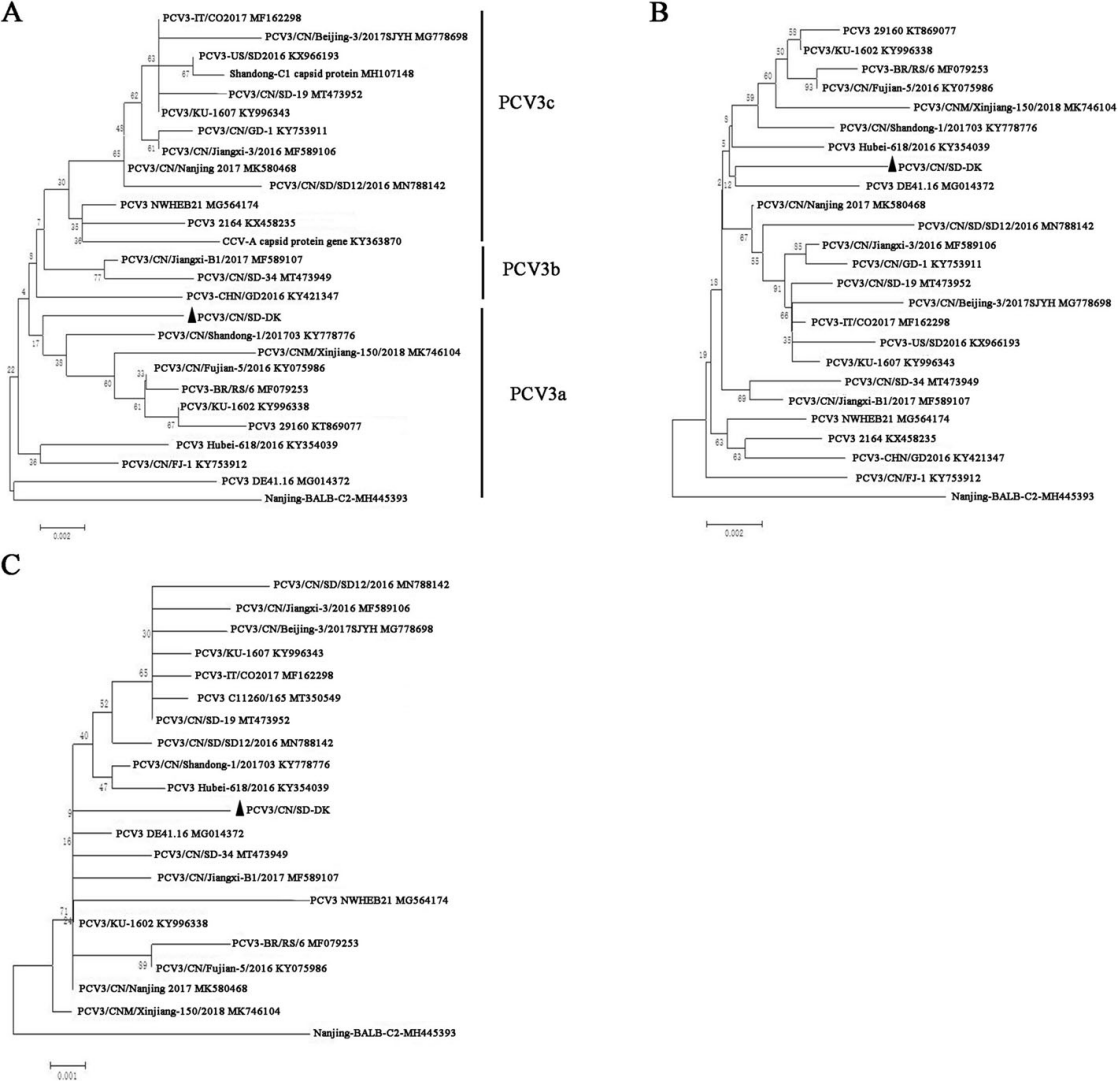
Phylogenetic tree based on ORF2 (**A**), complete genome (**B**) and ORF1(**C**) sequence of PCV3/CN/SD-DK in this study with that of representative PCV3 (dog, bovine, mice and pig) sequences was constructed using neighbour-joining method in MEGA6.0 software. PCV3 viruses can be divided into three subgenotypes. Each reference virus is presented by the virus name and GenBank accession number. Black triangles indicate the strain detected in this study. Bootstrap values from 1000 replications are indicated for each node. The scale bar indicates nucleotide substitutions per site

PCV3 can be divided into three major clades (PCV3a, PCV3b and PCV3c) based on the capsid gene sequences [15]. Considering the phylogenetic analysis of ORF2 nucleotide sequences, PCV3/CN/SD-DK was genetically related to PCV3 Shandong-1 201,703 strains originated from pig, which is described as part of the PCV3a cluster (Fig. 2A). However, the phylogenetic analysis based on of PCV3/CN/SD-DK complete genome (Fig. 2B) and ORF1 nucleotide sequence (Fig. 2C) were different from ORF2, and genetically related to PCV3 DE41.16 strain originated from pig, respectively. The gene sequences used in this study were listed in Table 1.

**Table 1:** Porcine circovirus 3 (PCV3) strains used in this study

Strain	Source	Country	Accession No.
PCV3/CN/SD-DK	Donkey	China	MW715784
IT/CO2017	Pig	Italy	MF162298
CN/Beijing-3/2017SJYH	Pig	China	MG778698
US/SD2016	Pig	USA	KX966193
KU-1607	Pig	SouthKorea	KY996343
CN/SD-19	Pig	China	MT473952
CN/GD-1	Pig	China	KY753911
CN/Jiangxi-3/2016	Pig	China	MF589106
CN/Nanjing 2017	Pig	China	MK580468
CN/SD/SD12/2016	Pig	China	MN788142
NWHEB21	Pig	China	MG564174
PCV3 2164	Pig	USA	KX458235
CN/Jiangxi-B1 2017	Pig	China	MF589107
CN/SD-34	Pig	China	MT473949
CHN/GD2016	Pig	China	KY421347
CN/Shandong-1/201,703	Pig	China	KY778776
CNM/Xinjiang-150/2018	Pig	China	MK746104
CN/Fujian-5/2016	Pig	China	KY075986
BR/RS/6	Pig	Brazil	MF079253
KU-1602	Pig	SouthKorea	KY996338
PCV3 29,160	Pig	USA	KT869077
Hubei-618/2016	Pig	China	KY354039
CN/FJ-1	Pig	China	KY753912
DE41.16	Pig	Germany	MG014372
CCV-A capsid gene	Canine	China	KY363870
Nanjing-BALB-C2	Mice	China	MH445393
Shandong-C1 capsid gene	Bovine	China	MH107148

## Discussion

Porcine circovirus 3 (PCV3) is a newly described member of the virus family *Circoviridae*, which was found to be associated with porcine dermatitis and nephropathy syndrome (PDNS), reproductive failure, and multi systemic inflammation [1, 16]. Recent studies have suggested that PCV3 was detected in non-porcine species including domestic hosts (mice, cattle and dog) and wild animals (wild boar and deer) [15, 17]. Recent years, mare abortion cause enormous economic loss in the donkey industry every year in China. Previous study suggested that EHV-1 and EAV could cause abortion of equine [18, 19], however, they are negative in present study. Interestingly, the infection of PCV3 in donkey with reproductive failure was firstly detected in our study. Therefore, we reported evidence of PCV3 in donkeys in China. However, the propagation and pathogenicity of PCV3 in donkeys have not been determined.

The genome of PCV3 is a 2000-nucleotide (nt) circular genome containing two major open reading frames (ORFs), ORF1 encodes a replication-associated protein (Rep), while ORF2 encodes the viral capsid (Cap) protein, that determines the antigenic characteristics of the virus [11, 20, 21]. PCV3/CN/SD-DK ORF2 gene is highly variable, shared 98.3-98.9 % nucleotide homology with other PCV3 ORF2, and 96.7–98.6 % at amino acid level. Phylogenetic tree suggested that PCV3/CN/SD-DK had a closer relationship with the PCV3 Shandong-1 201,703 strain based on ORF2 nucleotide sequences, while there are four variants of amino acids at 41, 56, 77 and 150 positions (Figure S1). However, the comparison of PCV3 Cap from dog, bovine and mice at aa residues 41 and 56 position have same discrepancy with PCV3/CN/SD-DK Cap (Fig. 1). However, effects of these mutations of the Caps on virus infections and pathogenicity are unknown.

## Conclusions

In summary, our study has provided first evidence of PCV3 frequency in donkey farm in China. The association of PCV3 with reproductive disorder in mares will be confirmed in the future. The genetic analysis and epidemiological investigation of donkey-origin PCV3 strains in this research will help enrich the data of PCV3 cross-species transmission.

## Methods

### Sample collection

With the permission of animal owners, we have collected 300 blood samples of 2–6 years old female donkeys with abortion and sterility (miscarring or mating more than once without pregnant) from 5 large-scale farms in Liaocheng city, China since Sep. 2019. The whole blood was collected into vacuum tubes with EDTA-K2, and stored at -80 °C until further analysis.

### Viral genome extract and detection

The viral DNA was extracted from blood samples with a Viral DNA Kit (Magen, China) according to the manufacturer’s instructions. In this study, the new primers of ORF2 gene (Table 2) were designed and used to detect PCV3 as described previously with modification [13]. Each qPCR reaction (20 µL) consisted of TB Green® Fast qPCR Mix (2×) 10 µL (TaKaRa, China) (the final concentration of mix was 1×), forward and reverse primers 1 µL (the final concentration of all primers was 0.2 µM) respectively, 2 µL of extracted blood DNA sample. Nuclease-free water was served as a negative control. The qPCR conditions employed the following cycling condition: 95 °C for 30 s and 40 cycles of 95 °C for 5 s, 60 °C for 15 s, and 72 °C for 15 s. The reaction efficiency and R2 of qPCR for PCV3 were determined with a dilution series of a plasmid (Cap gene cloned into pMD18-T) (Additional file 2, Fig. S2). The threshold cycle (CT) values determined from the plasmid dilution series were used to create a standard curve to determine the genomic copy number. Each assay was run in triplicates.

**Table 2:** The primer sequences used in this study

Primers	Primer sequences(5´-3´)	Length of product/bp	Reference
ORF2-F	ACGTCATCTCCGTTGGAACC	226	
ORF2-R	TGGAGCCAAGTGTTTGTGGT		
PCR1-F	ATTATGGATGCTCCTCATCGTG	553	(Wen, et al. [14])
PCR1-R	CATCTTCTCCGCAACTTCAGTC		
PCR2-F	GACTGAAGTTGCGGAGAAGATG	789	
PCR2-R	CGGCACGAAAGAAGTTTGGATT		
PCR3-F	CCCACATGCGAGGGCGTTTACC	895	
PCR3-R	CGAGGCCGCTTCATCATCCACT		

### Viral genome sequencing

One PCV3 genome sequence was amplified with three primer pairs of PCR (Table 2) as described previously [14]. PCR was performed using the following conditions: at 94 °C for 1 min, 35cycles of denaturation at 94 °C for 30 s, annealing at 56 °C for 30 s, and extension at 72 °C for 1 min, followed by a final step at 72 °C for 10 min. PCR products of three fragments were purified with DNA Gel Extraction Kit (TIANGEN, China) and subcloned into the pCE2 vector (Vazyme, China). PCV3 genome was determined and assembled using Sanger sequencing (Sangon Biotech, China).

### Multiple sequences comparison and phylogenetic analyses

The complete sequences of PCV3 strains were assembled using the SeqMan v7.1.0 program (Lasergene, DNAStar, USA), and sequences homology analysis was performed using the MegAlign v7.1.0 program (Lasergene, DNAStar, USA). The information of reference sequences were downloaded from GenBank database (http://www.ncbi.nlm.nih.gov/Genbank). Phylogenetic tree was constructed using the neighbour-joining method of MEGA 6.0 software. The bootstrap consensus tree inferred from 1000 replicates is taken to represent the evolutionary history, and the neighbour-joining method was used to infer the evolutionary history [22] .

## Supplementary Information

**Additional file 1.**

**Additional file 2: Figure S1.** Alignment of amino acid sequences of Cap. Alignment of PCV3 isolate PCV3/CN/SD-DK and PCV3 Shandong-1 201703 strain. **Figure S2.** Standard curves using plasmid DNA. Ct values were plotted against the log copy number of plasmid DNA. The regression curve (y), correlation coefficient (R2) and PCR efficiency (E) were calculated.

**Additional file 3: Table S1.** Copies number of PCV3 in donkey blood samples.

## Data Availability

All data generated or analyzed during this study are included in this published article and its additional files. The complete genomic sequence of PCV3 from donkeys generated in this study has been submitted to GenBank under accession no. MW715784.
