# Supplementary material for: First detection and phylogenetic analysis of porcine circovirus 3 in female donkeys with reproductive disorders
Source: BMC Vet Res. 2021 Sep 18;17:308. doi: 10.1186/s12917-021-03013-6 (PMC8449920; doi:10.1186/s12917-021-03013-6)
Supplement: Supplementary file 1 — Additional file 1. [file 12917_2021_3013_MOESM1_ESM.docx]

Porcine Circovirus 3 PCV3/CN/SD-DK isolate, Complete genome

TAGTATTACCCGGCACCTCGGAACCCGGATCCACGGAGGTCTGTAGGGAGAAAAAGTGGTATCCCATTATGGATGCTCCGCACCGTGTGAGTGGATATACCGGGCAGTGGATGATGAAGCGGCCTCGTGTTTTGATGCCGCAGGACGGGGACTGGATAACTGAGTTTTTGTGGTGCTACGAGTGTCCTGAAGATAAGGACTTTTATTGTCATCCTATTCTAGGTCCGGAGGGAAAGCCCGAAACACAGGTGGTGTTTTACGATAAACAACTGGACCCCGACCGAGTGGGAATCTATTGTGGAGTGTGGAGGCAGTATAGCGAGATACCTTATTATCGGCAAAGAGGTTGGAAAAAGCGGTACCCCGCACTTGCAAGGGTACGTGAATTTCAAGAACAAAGGGCGACTCAGCTCGGTGAAGCGCTTACCCGGATTTGGTCGGGCCCATCTGGAGCCGGCGAGGGGGAGCCACAAAGAGGCCAGCGACTATTGCAAGAAAGGGGGGGATTACCTCGAGATTGGCGAAGATTCCTCTTCGGGTACCAGATCGGATCTTCAAGCAGCAGCTCGGATTCTGACGGAGACGTCGGGAAATCTGACTGAAGTTGCGGAGAAGATGCCTGCAGTATTTATACGCTATGGGCGGGGTTTGCGTGATTTTTGCGGGGTGATGGGGTTGGGTAAACCGCGTGATTTTAAAACTGAAGTTTATGTTTTTATTGGTCCTCCAGGATGCGGGAAAACGCGGGAAGCTTGTGCGGATGCGGCTGCGCGGGAATTGCAGTTGTATTTCAAGCCACGGGGGCCTTGGTGGGATGGTTATAATGGGGAGGGTGCTGTTATTCTGGATGATTTTTATGGGTGGGTTCCATTTGATGAATTGCTGAGAATTGGGGACAGGTACCCTCTGAGGGTTCCTGTTAAGGGTGGGTTTGTTAATTTTGTGGCTAAGGTATTATATATTACTAGTAATGTTGTACCGGAGGAGTGGTATTCATCGGAGAATATTCGTGGAAAGTTGGAGGCCTTGTTTAGGAGGTTCACTAAGGTTGTTTGTTGGGGGGAGGGGGGGGTAAAGAAAGACATGGAGACAGTGTATCCAATAAACTATTGATTTTATTTGCACTTGTGTACAATTATTGCGTTGGGGTGGGGGTATTTATTGGGTGGGTGGGTGGTCAGCCCCCTAGCCACGGCTTGTCGCCCCCACCGAAGCATGTGGGGGATGGGGTCCCCACATGCGAGGGCGTTTACCTGTGCCCGCACCCGAAGCGCAGCGGG

AGCGCGCGCGAGGGGACACGGCTTGTCGCCACCGGAGGGGTCCGATTTATATTTATTTGCACTTAGAGAACGGACTTGTAACGAATCCAAACTTCTTTGGTGCCGTAGAAGTCTGTCATTCCAGTTTTTTCCGGGACATAAATGCTCCAAAGCAGTGCTCCCCATTGAACGGTGGGGTCATATGTGTTGAGCCATGGGGTGGGTCTGGAGAAAAAGAAGAGGCTTTGTCCTGGGTGAGCGCTGGTAGTTCCCGCCAGAATTGGTTTGGGGGTGAAGTAACGGCTGTGTTTTTTTTTAGAAGTCATAACTTTACGAGTGGAACTTTCCGCATAAGGGTCGTCTTGGAGCCAAGTGTTTGTGGTCCAGGCGCCGTCTAGATCTATGGCTGTGTGCCCGAACATAGTTTTTGTTTGCTGAGCCGGAGAAATTACAGGGCTGAGTGTAACTTTCATCTTTAGTATCTTATAATATTCAAAGCTAATTGCAGTTTCCCATTCGTTTAGGCGGGTAATGAAGTGGTTGGCGTGCCAGGGCTTATTATCCTGAGGGGTTCCAACGGAGATGACGTTCATGGTGGAGTATTTATTTGTGTAGTATGTGCCAGCTGTGGGCCTCCTAATGAATAGTCTTCTCCTGGCATAGCGCCTTCTGTGGCGTCGTCGTCTCCTTGGGCGGGGTCTTCTTCTGAATATAGCTCTGTGTCTCATTTTGGTGCCGGGC
