# Supplementary material for: First detection and phylogenetic analysis of porcine circovirus 3 in female donkeys with reproductive disorders
Source: BMC Vet Res. 2021 Sep 18;17:308. doi: 10.1186/s12917-021-03013-6 (PMC8449920; doi:10.1186/s12917-021-03013-6)
Supplement: Supplementary file 2 — Additional file 2: Figure S1. Alignment of amino acid sequences of Cap. Alignment of PCV3 isolate PCV3/CN/SD-DK and PCV3 Shandong-1 201703 strain. Figure S2. Standard curves using plasmid DNA. Ct values were plotted against the log copy number of plasmid DNA. The regression curve (y), correlation coefficient (R2) and PCR efficiency (E) were calculated. [file 12917_2021_3013_MOESM2_ESM.doc]

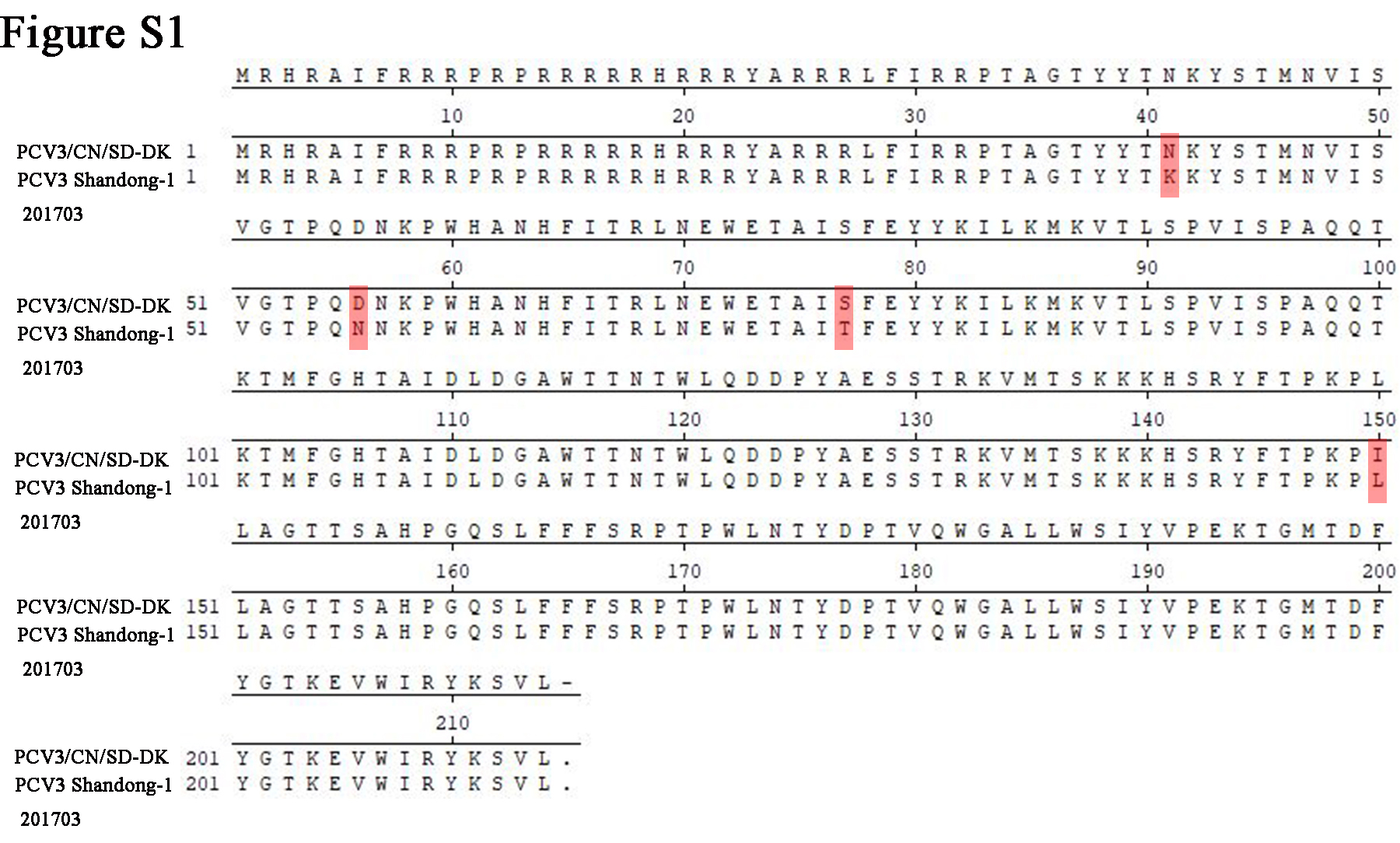


**Figure S1. Alignment of amino acid sequences of Cap.** Alignment of PCV3 isolate PCV3/CN/SD-DK and PCV3 Shandong-1 201703 strain.


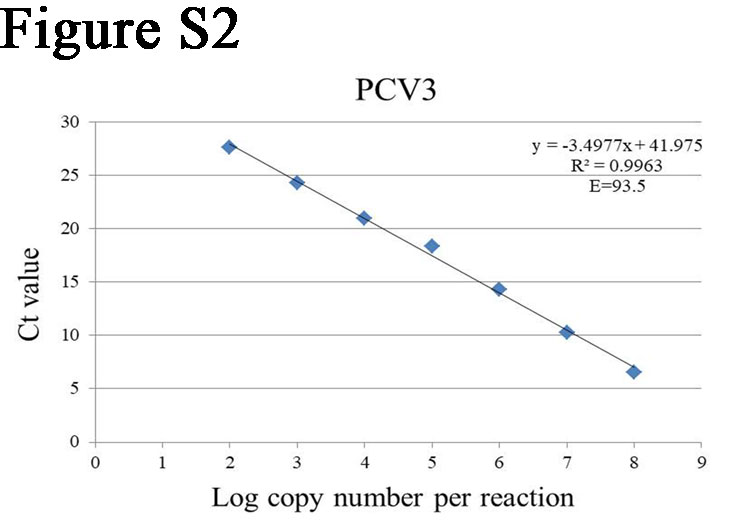


**Figure S2.** **Standard curves using plasmid DNA.** Ct values were plotted against the log copy number of plasmid DNA. The regression curve (y), correlation coefficient (R2) and PCR efficiency (E) were calculated.
