# Supplementary material for: First detection and phylogenetic analysis of porcine circovirus 3 in female donkeys with reproductive disorders
Source: BMC Vet Res. 2021 Sep 18;17:308. doi: 10.1186/s12917-021-03013-6 (PMC8449920; doi:10.1186/s12917-021-03013-6)
Supplement: Supplementary file 3 — Additional file 3: Table S1. Copies number of PCV3 in donkey blood samples. [file 12917_2021_3013_MOESM3_ESM.doc]

**Table S1. Copies number of PCV3 in donkey blood samples.**

| No. | Log copies number (mean ±S.D.) |
| --- | --- |
| | 3 | | --- | | 7 | | 8 | | 9 | | 13 | | 19 | | 22 | | 29 | | 31 | | 35 | | 36 | | 37 | | 42 | | 44 | | 45 | | 58 | | 67 | | 72 | | 75 | | 80 | | 82 | | 103 | | 118 | | 121 | | 135 | | 139 | | 143 | | 152 | | 155 | | 157 | | 171 | | 178 | | 184 | | 186 | | 188 | | 190 | | 195 | | 198 | | 204 | | 207 | | 209 | | 211 | | 215 | | 218 | | 226 | | 229 | | 231 | | 237 | | 239 | | 242 | | 244 | | 245 | | 251 | | 253 | | 262 | | 265 | | 269 | | 278 | | 281 | | 283 | | 288 | | 292 | | 294 | | | 5.68±0.01 | | --- | | 5.34±0.03 | | 5.56±0.05 | | 6.32±0.06 | | 5.94±0.05 | | 7.54±0.02 | | 6.37±0.06 | | 6.29±0.03 | | 5.36±0.02 | | 7.00±0.03 | | 6.05±0.01 | | 6.01±0.03 | | 5.92±0.05 | | 4.99±0.01 | | 5.33±0.01 | | 5.65±0.02 | | 5.52±0.01 | | 5.53±0.02 | | 5.07±0.03 | | 5.41±0.04 | | 5.28±0.05 | | 5.14±0.06 | | 5.05±0.02 | | 4.99±0.06 | | 4.35±0.10 | | 4.36±0.14 | | 4.71±0.13 | | 4.57±0.10 | | 4.21±0.03 | | 5.20±0.04 | | 4.75±0.01 | | 4.81±0.05 | | 4.40±0.03 | | 5.06±0.05 | | 4.68±0.03 | | 4.97±0.08 | | 5.88±0.04 | | 5.94±0.02 | | 5.61±0.04 | | 4.90±0.06 | | 5.23±0.03 | | 6.40±0.02 | | 6.10±0.07 | | 6.48±0.02 | | 5.25±0.02 | | 4.45±0.02 | | 5.35±0.07 | | 5.25±0.06 | | 5.69±0.05 | | 4.94±0.03 | | 5.28±0.03 | | 4.66±0.08 | | 6.99±0.05 | | 5.51±0.05 | | 4.70±0.01 | | 6.75±0.03 | | 5.94±0.07 | | 5.22±0.04 | | 4.41±0.06 | | 5.02±0.04 | | 5.54±0.04 | | 5.02±0.02 | | 6.97±0.04 | |

Viral DNA was extracted using viral DNA kit, PCV3 viral copies were caculated by qPCR in donkey blood. Data are presented as the Log copies number (mean±S.D.).
